# Phototoxicity – from molecular descriptors to classification models

**DOI:** 10.1186/1758-2946-3-S1-O10

**Published:** 2011-04-19

**Authors:** Friedemann Schmidt, Andreas Evers, Alexander Amberg, Gerhard Hessler, Catherine Robles, Karl-Heinz Baringhaus

**Affiliations:** 1 Sanofi-Aventis, R&D Frankfurt, Germany; 2 Sanofi-Aventis, R&D Montpellier, France

## 

Potential photoactivation of certain pharmaceuticals, cosmetic ingredients and natural products by sunlight (e.g., phenothiazines, arylsulfonamides, or coumarins) has to be considered early on in development in order to avoid serious adverse effects (for example phototoxic or photoallergic reactions). Current clinical trial registration guidelines (FDA May 2003 [1], EMEA Dec. 2002 [2]) recommend photosafety testing of molecules if they exhibit strong absorption bands between 290-700nm and if they are significantly partitioned in human skin or eyes.

The UV absorption coefficients and the tissue partitioning of a compound are considered as important factors for phototoxic effects. However, the rationalization and prediction of phototoxicity by (quantitative) structure-property relationships ((Q)SPR) offers a valuable strategy to reduce experimental testing if an appropriate precision level of the underlying model is guaranteed.

A diverse data set of known phototoxicants and non-phototoxicants including various molecular chemotypes (90 % of them are pharmaceuticals) was compiled. After geometry optimization the maximum absorption wavelength of each compound was calculated by semi-empirical methods followed by subsequent computation of molecular descriptors. Our *insilico* analysis (e.g., PLS and recursive partitioning) of quantum chemical as well as classical molecular descriptors (e.g., LUMO, HOMO/LUMO gap, electron affinity, ionization energy, molecular fragments, physicochemical descriptors such as logD, p*K*_a_ and logP_eff_) has led to predictive photosafety classifiers. Model validation was performed with a proprietary external test set of an *in vitro* photosafety assay (3T3 neutral red assay).

Our photosafety models are currently applied in a prospective manner in the prioritization, classification and labeling of newly designed molecules.
